# Editorial for the Special Issue on Plant Biostimulants in Sustainable Horticulture and Agriculture: Development, Function, and Applications

**DOI:** 10.3390/plants13172342

**Published:** 2024-08-23

**Authors:** Mohamad Hesam Shahrajabian, Spyridon A. Petropoulos

**Affiliations:** 1 National Key Laboratory of Agricultural Microbiology, Biotechnology Research Institute, Chinese Academy of Agricultural Sciences, Beijing 100086, China; 2 Department of Agriculture, Crop Production and Rural Environment, University of Thessaly, 38446 Volos, Greece

The growing need for food production through sustainable cultivation practices, without reducing crop yield and producer income, is a major objective due to increased environmental pollution and the gradual degradation of cultivated soils [1,2]. Various components with bioactive characteristics can be used as biostimulants to boost plant growth and development under normal and stressful conditions [3,4]. So far, six distinct categories of biostimulants have been recognized, including microbial inoculants; humic substances, such as humic acid and fulvic acid; protein hydrolysates, and amino acids; biopolymers; inorganic compounds; and seaweed extracts, all of which are commercially available with wide applications in agriculture [5,6]. The most important biostimulant effects on crops are the acceleration of crop establishment, the improvement in nutrient uptake and nutrient use efficiency, the induction of tolerance in nutrient leaching, the improvement in root development, the removal of heavy metals from contaminated soils, the improvement in crop performance, the stimulation of the immune system of plants, the improvement in the visual quality of final products, and the induction of the biosynthesis of plant defensive biomolecules. Apart from single-product effects, there are many reports that show the combination of biostimulants results in beneficial effects on agricultural and horticultural crops, which are usually better than those of single biostimulants. Different classification approaches have been proposed so far, based either on the origin of each biostimulant, such as biological or non-biological, microbial and non-microbial, or on the mode of action which divides biostimulants into phytohormonal and non-phytohormonal ones [4,5,6]. Biostimulants are components which can increase plant growth, but do not qualify as essential plant nutrients, while biofertilizers are live microbes whose primary impact is to increase plant growth. It is important to consider the point that the basic difference between biofertilizers and biostimulants is that biofertilizers contain different nutrients, while biostimulants do not have the plant nutrients. Microbial biostimulants can synthesize IAA, which can promote root branching and plant growth of the plants under biotic and abiotic stresses, and they can also alleviate salt stress as they are associated with a high level of IAA and can enhance them in the re-establishing of favorable water potential gradients under water shortage conditions, as well as increasing film hydration around the roots. This Special Issue provides different examples of agricultural and horticultural plants which have been influenced by various kinds of biostimulants, while considering different mechanisms of action. Considering the above, new biostimulant products need to produced and applied; however, this should be realized under a new approach focusing on the synergistic impacts of different biostimulatory agents instead of single-product application.

This Special Issue focuses on the functions and roles of various types of biostimulants in different horticultural and agricultural crops within the framework of sustainable crop management, aiming to gather critical and important information regarding their positive effects on plant growth and final yield, as well as regarding their impacts on the quality of the final products. Furthermore, the major limitations of these practices as well as the future prospects of biostimulant studies will be presented. Furthermore, we are grateful to the reviewers for dedicating their time and maintaining the standards of the publications included in this Special Issue.

## References

[1] Shahrajabian M.H., Chaski C., Polyzos N., Tzortzakis N., Petropoulos S.A. (2021). Sustainable Agriculture Systems in Vegetable Production Using Chitin and Chitosan as Plant Biostimulants. Biomolecules.

[2] Shahrajabian M.H., Chaski C., Polyzos N., Petropoulos S.A. (2021). Biostimulants Application: A Low Input Cropping management Tool for Sustainable Farming of Vegetables. Biomolecules.

[3] Sun W., Shahrajabian M.H., Kuang Y., Wang N. (2024). Amino Acids Biostimulants and Protein Hydrolysates in Agricultural Sciences. Plants.

[4] Sun W., Shahrajabian M.H., Soleymani A. (2024). The Roles of Plant-Growth-Promoting Rhizobacteria (PGPR)-Based Biostimulants for Agricultural Production Systems. Plants.

[5] Shahrajabian M.H., Petropoulos S.A., Sun W. (2023). Survey of the Influences of Microbial Biostimulants on Horticultural Crops: Case Studies and Successful Paradigms. Horticulturae.

[6] Sun W., Shahrajabian M.H. (2022). The Effectiveness of Rhizobium Bacteria on Soil Fertility and Sustainable Crop Production under Cover and Catch Crops Management and Green Manuring. Not. Bot. Horti Agrobot. Cluj-Napoca.

